# Supplementary material for: Expression Profiling Reveals the Possible Involvement of the Ubiquitin–Proteasome Pathway in Abiotic Stress Regulation in Gracilariopsis lemaneiformis
Source: Int J Mol Sci. 2023 Aug 1;24(15):12313. doi: 10.3390/ijms241512313 (PMC10418974; doi:10.3390/ijms241512313)
Supplement: Supplementary file 1 [file ijms-24-12313-s001.zip › ijms-2474555-supplementary.pdf]

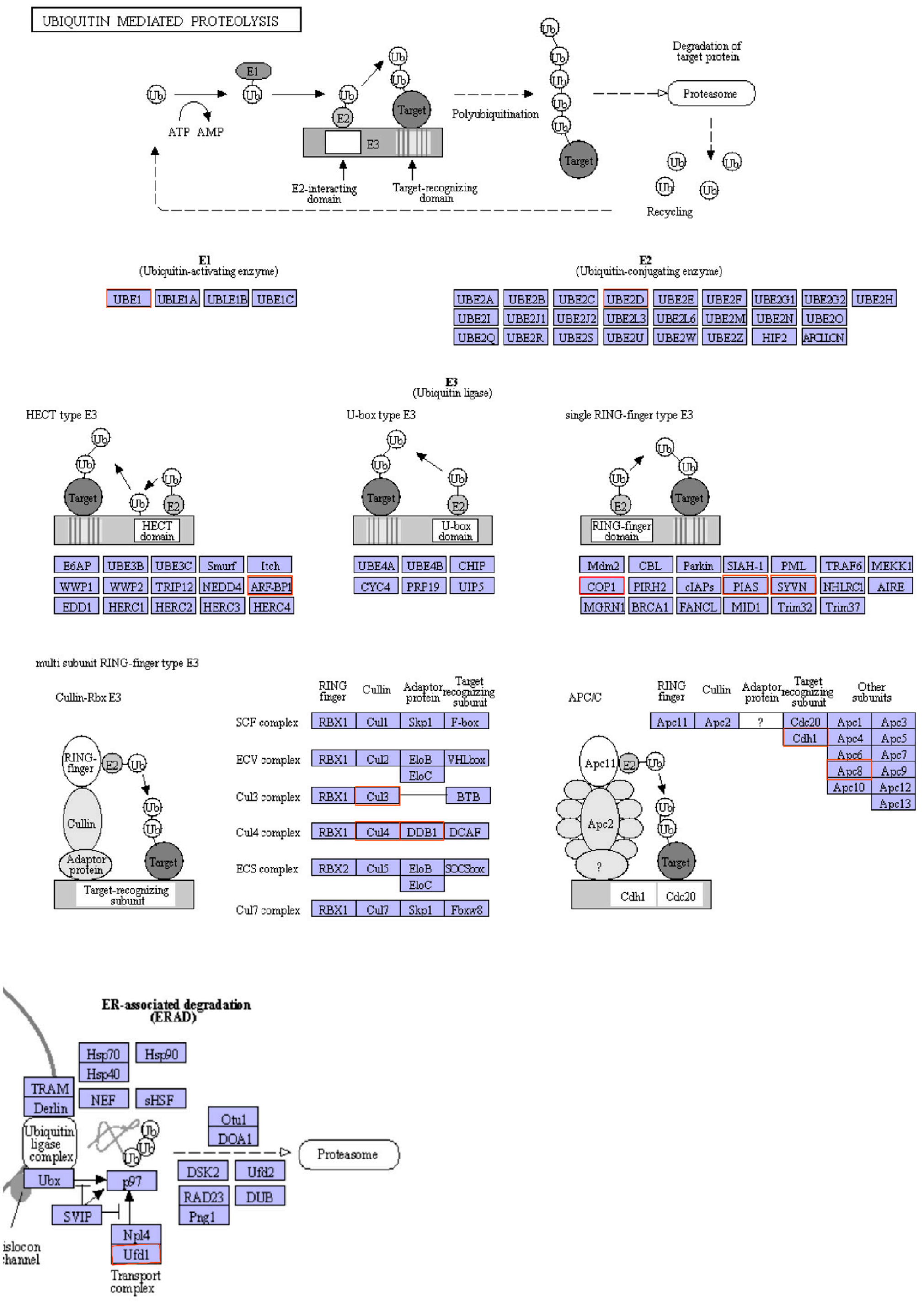

Supplementary Figure S1. The ubiquitin mediated proteolysis pathway. The 12 key up-regulated genes are circled in red boxes.

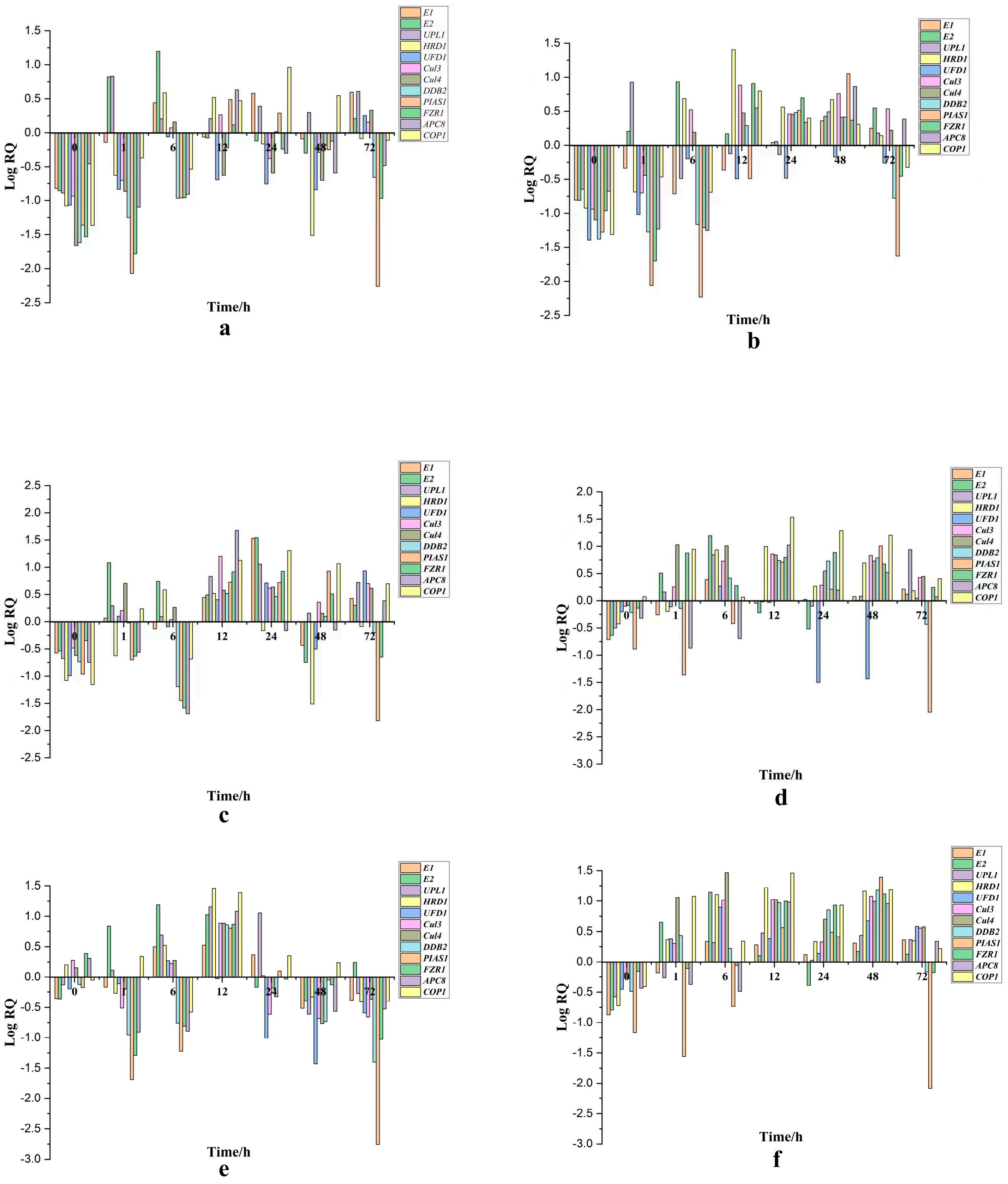
E1
E2
UPL1
HRD1
UFD1
Cul3
Cul4
DDB2
PIAS1
FZR1
APC8
COP1
Log RQ
Time/h
0
1
6
12
24
48
72
a
b
c
d
e
f

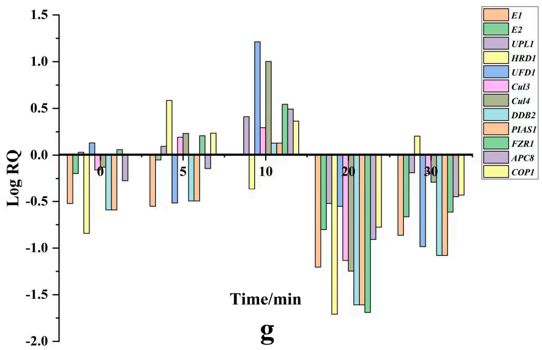

g

Supplementary Figure S2. The qRT-PCR analysis of UPS genes. **(a–g)** represents the expression levels of different genes under high temperature, low temperature, O_3, 5% PEG, 10% PEG, 20% PEG, and water shortage respectively. The horizontal axis represents the time of treatments, and the vertical axis represents Log RQ. "RQ" represents $2^{-\Delta\Delta Ct}$, "Log RQ > 0" means the gene is up-regulated, "Log RQ < 0" means the gene is down-regulated. The column of different colors represents different genes. The transcription of each gene in the control group at the same time point was used as a calibrator to determine the expression level of genes in the treatment groups.

Supplementary Table S1. Genes up-regulated in response to different abiotic stress treatments.

**Stress treatment and time of exposure**	**Total no. of genes up-regulated**	**Genes up-regulated**
High temperature-1h	2	*E2 UPL1*
High temperature -6 h	6	*E1 E2 UPL1 HRD1 Cul3 Cul4*
High temperature -12 h	7	*UPL1 HRD1 Cul3 PIAS1 FZR1 APC8 COP1*
High temperature -24 h	5	*E1 UPL1 DDB2 PIAS1 COP1*
High temperature -48 h	2	*UPL1 COP1*
High temperature -72 h	6	*E1 E2 UPL1 UFD1 Cul3 Cul4*
Low temperature-1h	2	*E2 UPL1*
Low temperature-6 h	4	*E2 HRD1 Cul3 Cul4*
Low temperature-12 h	8	*E2 HRD1 Cul3 Cul4 DDB2 FZR1 APC8 COP1*

Low temperature-24 h	10	*E1 E2 HRD1 Cul3 Cul4 DDB2 PIAS1 FZR1*<br>*APC8 COP1*
Low temperature-48 h	11	*E1 E2 UPL1 HRD1 Cul3 Cul4 DDB2 PIAS1 FZR1 APC8 COP1*
Low temperature-72 h	7	*E1 E2 UPL1 HRD1 Cul3 Cul4 APC8*
O_3-1 h	7	*E1 E2 UPL1 UFD1 Cul3 Cul4 COP1*
O_3-6 h	5	*E2 HRD1 Cul3 Cul4 UPL1*
O_3-12 h	12	*E1 E2 UPL1 HRD1 UFD1 Cul3 Cul4 DDB2 PIAS1 FZR1 APC8 COP1*
O_3-24 h	10	*E1 E2 UPL1 UFD1 Cul3 Cul4 DDB2 PIAS1 FZR1 COP1*
O_3-48 h	7	*UPL1 Cul3 Cul4 DDB2 PIAS1 FZR1 COP1*
O_3-72 h	8	*E1 E2 UPL1 UFD1 Cul3 Cul4 APC8 COP1*
5%PEG-1 h	6	*E2 UPL1 Cul3 Cul4 FZR1 COP1*
5%PEG-6 h	10	*E1 E2 UPL1 HRD1 UFD1 Cul3 Cul4 DDB2 FZR1 COP1*
5%PEG-12 h	8	*HRD1 Cul3 Cul4 DDB2 PIAS1 FZR1 APC8 COP1*
5%PEG-24 h	9	*E1 HRD1 Cul3 Cul4 DDB2 PIAS1 FZR1 APC8 COP1*
5%PEG-48 h	11	*E1 E2 UPL1 HRD1 Cul3 Cul4 DDB2 PIAS1 FZR1 APC8 COP1*
5%PEG-72 h	10	*E1 E2 UPL1 HRD1 UFD1 Cul3 Cul4 FZR1 APC8 COP1*
10%PEG-1 h	3	*E2 UPL1 COP1*
10%PEG-6 h	7	*E1 E2 UPL1 HRD1 UFD1 Cul3 Cul4*
10%PEG-12 h	11	*E1 E2 UPL1 HRD1 Cul3 Cul4 DDB2 PIAS1 FZR1 APC8 COP1*
10%PEG-24 h	6	*E1 UPL1 HRD1 COP1 FZR1 PIAS1*

10%PEG-48 h	1	*COP1*
10%PEG-72 h	1	*E2*
20%PEG-1 h	7	*E2 HRD1 UFD1 Cul3 Cul4 DDB2 COP1*
20%PEG-6 h	9	*E1 E2 UPL1 HRD1 UFD1 Cul3 Cul4 DDB2 COP1*
20%PEG-12 h	12	*E1 E2 UPL1 HRD1 UFD1 Cul3 Cul4 DDB2 PIAS1 FZR1 APC8 COP1*
20%PEG-24 h	11	*E1 UPL1 HRD1 UFD1 Cul3 Cul4 DDB2 PIAS1 FZR1 APC8 COP1*
20%PEG-48 h	12	*E1, E2, HRD1, UFD1, UPL1, Cul3, Cul4, DDB2, FZR1, PIAS1, APC8, COP1*
20%PEG-72 h	9	*E1 E2 UPL1 HRD1 UFD1 Cul3 Cul4 APC8 COP1*
Water shortage-5min	6	*HRD1 UFD1 Cul3 Cul4 FZR1 COP1*
Water shortage-10min	10	*E2 HRD1 UPL1 Cul3 Cul4 DDB2 PIAS1 FZR1 APC8 COP1*
Water shortage-20min	0	
Water shortage-30min	1	*UFD1*

Supplementary Table S2. Formulation of stock solutions for f/2 seawater medium

f/2 solution	
Name	Dose
$NaNO_3$	7.5 g
NaH_2PO_4	0.5 g
ddH_20	100 mL

f/2 vitamin solution

Name	Dose
Vitamin B_1	10 mg
Vitamin B_{12}	0.05 mg
Vitamin H	0.005 mg
ddH_20	100 mL

f/2 trace element solution

Name	Dose
$FeCl_3 \cdot 6H_2O$	315 mg
$CuSO_4 \cdot 5H_2O$	1 mg
$MnCl_2 \cdot 4H_2O$	18 mg
$ZnSO_4 \cdot 4H_2O$	2.2 mg
Na_2EDTA	416 mg
$CoCl_2 \cdot 6H_2O$	1 mg
$Na_2MoO_4 \cdot 2H_2O$	6 mg
ddH_20	100 mL

Add 1 mL of each of the three solutions mentioned above to every 1000 mL of sterile seawater.

The following are the putative promoter sequences of related genes

E1

1 AGTCTTGAAT GCACGCATGC GCTCAGTATG TAACAATCGG TGCGTGAAGT TGCGACTTGC

61 CGATATTCTC TGTTGGGACT TTCATCCTGA GTCCAACTGA CAGCGGAGCA AATTGAACGA

121 GCATGCTGCA CATAGCCGGG AAAAAAAAGC CATGAACAGA GATCTCAAGT TGGAAATGTT

181 TGCCCCAACC ATTCGGGTGA TGATCATTTG TCTTGTCTCA CATTCAGCAG CATTCCCATG

241 AAGTGCCGAT TCTTGGGTTC CATCCACAGA CGCAGGTACC AGGCGCCAAC GTATGTTTGA

301 TGCCCAGCCC GACACCGTTG AGGTAGCTGG ACCGCCAGAA GCGATTTTCA ATGATAGCAT

361 ATGGCAACCG GACTCCTCCA GTGGAATCGC GTCACCGCCG CTTTGGAAGT AGTAGCTAAG

421 CGAACCTCGG ACCCGGGCCT GATTAGGACT TCTTTTTCAG TACGCATTAC AAATCGGTAT

481 CGATTGCATC CTTCAAAGCA ATATACAGCA AGCGCAGCAA CTCGTGCTCC CTCCATGGAG

541 GCGGTGATTA GCTTTTCGGG TAACCCAGTC CCAAGCAATG ATGGCACGTG GGAGATTACG

601 TTCTCCTTGA GCATTGCGGT TGAATCTGTG ATAAATGTTT ACACCCTCGT TGATCCATTA

661 CTAGCCTATG TTTGAATAGA AAACAAGCTG TCGAGGCCAA TCTGCTAAAC ACTCACAACG

721 TTGTTGAAGC TTTGATAGAA ACGATTGCGT TGTGAGAGTT GGGCAGCATT GCAGAGAAGG

781 GTTCAGACTT GGTCTCCAAT AACCACAACT CTCGATTTTT TTAAACAAAA TATGAATTCT

841 CATGGCAATG CCTTTCGTAG GGAAGGAACA GCAGTTCGTC TATGCGGTAC GCGGGCTTGA

901 TCCCAACCGT GTATCAAAAG CGCCGCTCCT CCCAATTCGT CTACTACCTC CCGGGAATCT

961 GCAGCCTAAG GCTTTATTTC TCAATCACAA GACCTCAACT

E2

1 AGTGGCGAAG GTTGCACGAG AATACACCGC CGCGTCAGGG CATACGCTCA TTTGCTTCTG

61 ATGTAGTACA TCTCGGAACG TTCGGATTTG CCAGCTCTAA AATTTGGCGA AGCCGAAGAG

121 GACTCCAGTT CGCAGCAACT TTGGTTTGCT CTTCACTTTG CTCACAGTTT TGCATTGCGA

181 CTCTCACCTC ACTGTTCTTC ATTCTTTTCA AGCGCACAGC GGGAACAGGG CCAGCTACCA

241 AAAGCTACTG GAGCCCATGA AAGCAAACGT TCCGCACTTG CCCTAGAATT GGTCGGTCAT

301 CTCGAACTTC GTTGTATATA GACACTCATG AATCGATGGA CTCCACGCTT GTCGAACAAG

361 TACGGCAGGG AGTTCCCAGA TTGAAGTGTC AGGGCCCCAC CAAACGGGGC AAAACAGTGA

421 GAGTAGCACA CACTGTTGAG TCAACAGTAT TGACGTTGTG AAACACGACC CACTCGGTCC

481 AAGACTGTCT GCGGACCAAC AGTTCAGTTT CGAGCGTCCT GTAATTGTTA CTCGGAAGGG

541 GCAAAGGTTT TCGTCCAAGT TGAAGGTTTT TGGGCCCAAT TCGGAACAAA TTTGTTCGAA

601 GCGAAAAGTG ACGGAGGCTC CTCCGCTCTG ACGTTGCCAC GCAGCAGAGC TCTCGGCATG

661 CGAAGTTTCT CGACATTTGC TCCCCAAAGT TGCAATTTGT CTCTGCCGCG TGTAAGCAGT

721 GGGTCCAAAG GTTACAATTT TTCGCTCTTG CAGTCGAACG CTCGTCACAC GCGTACTTGA

781 AGCCCTTCGC TGACCTCTCC TGCTTTGGTC GGCTTCCACG GACAAACGCC CTCATCTTTT

841 GTTCTACCAC TTTCTTGCTC TACTAAAGGC TCTTCTTTCG CGGTCTTCGA ATTCGTTCTT

901 CGGGAGCTCG ACGTATCACG AAAGATTACT CGAGGCCACT CTTCACCCAC GCCTGTCTAC

961 CCACTTAAGT TTCAACCCGT TTGTAACACA AGTAGTACCG

UPL1

1 TGTATTCAAA ATCCGCGGTT GGAACGACTT GCCAGGGCCT CGGTGACAAC GTTCATGGCG
61 TCAATGAAGC GGAAAAGGCA CCTGCACAGC AGAAGACAGG AGTGCGTGAT AGGCTGTTAG
121 AGATGTCAGT GGGATTCGAG TGCTCAGGGA TTAACATACT TTTCAACGCA GTCAGTTTCT
181 CGACTCGACA TTTGCTTGCG AGAAACATCT GCACAAACGT TGAAGCACTT ATCAGTAACT
241 TGCTCCCGAA AGACTTCGCC CATCTGAGCC TTGACTTGTG CTTCCACACG AGCCATCATT
301 TCGGTAACGT CCATGTTTCC TGCAGACATA ACGCCTATGA ACCGAAGCCT AAACCTTGCT
361 GCGAATGAGA ATGAATGAGC AGTGTCGGGT CTTGGTGATC AGCAGCAGTG AAGCGCAACC
421 GTGATGCCAA AAAGGGTGAA GTAATACGGT TGGCAATCGA TTGCTACGCG GCAGGCCTCC
481 ACTCGAGATA ACGCCGGTTT CAGGGGCTTT CGGCGGTGGC TGTCCGCCGG TACACGTAGC
541 TGCTTTTGAG AAGCGAAGTT GCCGTTTTGT TCGTGGACGT GGATTGTAAA ACGGGAAGAC
601 TCAATTTCCA GACCCCACTG GCCCGTGTCA TACGTTTAAG CGTTGTCCAG GTCTCCATAA
661 CGCCTCGAGA AGCCTGCCTG CCTGACGCTC ACTGCCCTTT TCACAGCGTG ATGATCTGTG
721 GCATGTGGGA TGTCCGAGGC ACCCTGGAAG TTCCGCCGGT TCTGACCAGC TGCGCTCATG
781 CGGATCGAGT CGCGCGCACA CCCTCCACCT CTTCACATTG TGTTCCGAAA GTGCCGCAGC
841 ACACCTCCAA CATCAGCGTC GAAACGAAAC AGCCCTTATA CTGCTCGCCA CCGTGTCTTG
901 TTTCGTTTCA CGACCGCGAC TCCTAACTCG CCCAGGCCTG TTTCAAGAAG GGGACATTGC
961 ATATACGACC CTTCCCCTGT AACACTTCAC TAACACCCCT

HRD1

1 TCGTTTGCTG CGGGAGCCTT AGCGAGTGAG CGTCGAGCAT GCGAACGTCC CGAGTTTGGT
61 CGCACAAACC TACTGAACAA GCTATCCAGC AGCAATTCGG GCATCCGCGC ATAAACCGGA
121 CTACATAGCA GAAGCAGCAA CAGGAGCAGG CAAAGGATGC GGGGCAAGGG CATGTCGGGG
181 AGGAAGCAGT TGCCGAGAGT AGCGGCTTCG GGAGACGGAC AGCCCACTGG TCCGGTGCGA
241 CTGTGGAGTT TGGAATTGCA ACTGATTCAT TGTTAGAGTC GTTTGACTAA TTGAGCTTCG
301 GTCAGCACTC CGTGCGTACA GAGAACCGCT CGCCGTGGCG GCAGTTTTCA ATCGTCATCC
361 TCCAGTTCGG TGAAACGCAA AGTAGATACC CTTGTACTGT CCCAACGTAA ATTTTCCGTC
421 TCCCTGTAGA GGCGAAGCAG TCATCGAAAC GGTTATGAGA TGTACCTCGT GCTGGGATCG
481 CGGTCTTGCG TTTCGCTCTT CGCGAAGACA TCACAGGGCT GCTGTCGCAG TCTCTTCAAC
541 AAGGATTCCG CTCAGCCACC TCTAGCTAGA GTGCCTCCAT CCCCGCAACT TTCATACGAA
601 CACGTTGCAG CAGTTTCCAG TTTTTAAGGA TGAGTTATAG GGGATGACTT GACTTTGCTG
661 TGCTGTAGCC TGTATCGGCA GTGGGCCAAT AAAGGACAGT CAACTTTCCA AGAACGGATG
721 TTCATTGGAC ACTTGTTGCT TGTGGTAGAT TCTACAAAAA ACAGCTCTCA AGTATTTTTC
781 TTCCCGCACA ACCTGGAACC AGAGGTGCCT CTTATCGTTC TCAACCAGTC TTGTCCCACA
841 TCACAACAAC TCCCAGATGG TCACATCGCT GATTTACTTA CCACACTGAG ATACTTATCC
901 CCGCGCTTGG CTAACGGGCG CTTGTATTTT CAACCAAACC CTTCCCCCTC TTTCATCTTA
961 ACTTACTTGT ACCATCAAAT AGTTCTACGC TACTTCCAAC

UFD1

1 GAAGATGGAT ATACGCCATC AGCACCATCC ATTGATAGGG CAGTGGTGCA GTGTTCTGAG
61 AATTGTTCAG AATGGAAAGG CGGACAAAGG TATCTGTAAC AATCAAAGAG CACAACGTAG
121 AACAAAACAT GTGTGTTGGT ACTACCTGGT GTCAGTTTCT ACTAGAGCCG CCGTCCGCAG
181 AGAAAACGCA TGATGTGCAG TTCTGATTCT TTCAGAAAGT TGTTGCATTG TGTAAATTCG
241 TCATAGCCGA GAGACTAGGA CTGCCTTGAA GGCGTCAAAT ATCGTCCTTA AGGAACAGAA
301 CAGAACGTGA TCGCGCCAGA ATACGATCTG GAAGAACTTA CGCGTAGTCA AGCTCAAACT
361 TCACGGAGAC TTGTGCCCTT GGAGGCAAAC TGAACGGCCT GGAGGCTCGG CCCTGAAGTT
421 GAGCGGTGAT AGCTGCCGGA AGGGATCAAA CTTGCCGTAC CGTTGATAGT CTTAGACCAC
481 CTGTATTGAT AACCAGTGCA TCCGGCGTTG AAGCTGGCGC GTTTGAGCGT GTTCCACGTG
541 TGGCCGTGTC CACGCGTCCA GCTGCCAACG AAGAGCTGAG GAGAACGGTA CGACGTGGTC
601 CCTTTGGGTA CAGTTTGTAG TACCATACCT GGATCCATGT ATGAAAGGGC GAGTTGTGCC
661 GGATCCTTTT CGTGTTTCGT TCCGCGATAC ACGTTTTGCG TTCCACAACC ACTGCCATTC
721 CGACGTTCAA CTGAACACAC CCCTGCTCGC AGACCAAATG CAGCCGTTCC GCTGTTGCCC
781 AAACACCTCA ACACCGCAAC GTTGTGAACT TCGGGCCTCG CCAAGTGAAG CACAGCGCCT
841 CCTTCCTGCG ACCGGCACGC ATCCGCGAAC ACATCTATCC AAGCAACAGA TCTGCCATCA
901 ACCGTTTTCC CAGCACCAAT CCGTCGAGCT GTCTCCAATT TCGGAGTCTG CTTGAGAACC
961 TCGCACCAAC GTCGCTACAC ATTTTCCCTC TTGCTACACC

Cul3

1 TCAGCAGTGG GTGAGGGGTG AGAGCACAGA ATATTGTGCG ATTTGAGCGC GATGCGTTCC
61 GTTTTAGACC CCATGGAGGC GATATGAAGG TGTGAGTGGA GCAAAAGTCG TGCTGGAAAA
121 AGGAGCGGCA CATGACGAAT TGCTGTTTTC GTCAATTCAT TGATGTGCCA GCCGTTATTT
181 TGCTAACCCC TTCACCGTTC GCACTTGACC GCACGCAGTC GTGGCGGTCG CCGACGTACC
241 CGCACGCAGT TGGCAGCCAA ATTGGAACAA TTGATACCAA AAGCGACGAG ACAGCTGGTG
301 GACAATCGGG ACCGTCACAG GGACTTGAGT GGGGAAAGCG GGGGGAGTGG ACACGACATT
361 CATCATTAGG CTGGATACAT TTTTTTTAGT AGTAGGGCTG CTGCCGAGAT CGCAGCATGG
421 CATTGCTCAC CGGTCCAGAG GAGGCTTCAC GTCGAGAATC GCTGTGCATC ATTGAGGCCC
481 GTTCGGGTGT AGTACGCCAG CTGTGCGCAT TCCGCGCAGC GCTGGACGGT TACTTCAGCG
541 CACTGACCAC GTGTATGCGT AGCGCACGTG CATCGCTCCG CCATTCGTCC AATCAAACGG
601 CAGCGACACA TGAGCAGTTT GATTTGAGGC TTCGGCAATA CACTCGAGAG GCTACTCGCG
661 AGAACCGGGC CAAGCCCACA GCGAAACGAA CCAGCGTGGA CAAGCGCAGT TTGCGGTTTG
721 TCGCATAGCA TACCGCGTTA CACGCCGCCC TTGGAATGCA CGCACGTCCT TCTTCACAAC
781 CCCTCTTCGT GGTCAGCACT ACTCTCCGAC GCCCTTAACT CGCTGTGTCC CTTTCGCCTG
841 TTTTGCCGGC CTGTTTGCTC TTGCTTACAA GCACAAAGGT GGTCTAGCAT ACCCACAGCC
901 CAGCCCCTTC CGCTTTCGTT CAACCAGCCT GTAATTGCAT CCAATTTCCT CACCGTTCTC
961 TCTCTTGCTC TACGCGCAGC AACCCCACCC CCTCTGCATA

Cul4

1 CGCGCAGCGA GTTGAGCTTG GAGGTGGAGA GCTCGAGGCC CATTTCAAAG AGGAAGAAAA
61 CGACGCCCAG TTCGGCCAGC GCCTTTGTGG TACGCACGTC GTTGACGAGG GCAAAGGCAT
121 TGGGGCCTAG CGCCACGCCC GCGGCCAGGA AACCCAGAAT GGGAGATATT CCAAAACGAC
181 GCGCAAGCGG GATGATAAGC ACGTGGAGAT GAGGCACACC AGGGCGTCAT TGACGGCGTG
241 CTGATGGGAG TGATCGTCGC CATGGTCGGA ACGAGTCAAC AAAGATTGAC GAAGACGCTC
301 TACCCGACGA AGCAGGCGGC GGCAGGAGAG GGGGGGATGA GCGGGGGGGT GGACGGGAGC
361 GGGTGGGTGC GGACGCTGGG TGGGGCGTTG CGGCGTGACG GCGATGGCGG CGGGACGGGA
421 ACGGCGACGC GGGTGAGTGG CCGCAAGGGC CGAGGCGGAA GAAGGCGGGG ACGACGCATC
481 GGGAAGCGCG CGCAAGGGGG CGGGGCGAAG GAGCTGAGCG GCGAAGGCGG CAATGGCAAG
541 GGCTCGACGC CACACGGGCG GGGGGCTGCG CTTCGGGGGC GGATCGGCTG CGACGGCGCG
601 GGGCAGCGAC CGGGCGGCGA GCGGGACGCG GGGGGAGGCG CGACGAAGTC GACGAGGCGG
661 AAGCGCGGCG ACGAACAGCG CGGGACGCGC GGTGGCGGGC GGCATGGCGG CGACGAAGCG
721 TAACGGAGGC GGGTGGCGGA GCGCTCTCGC CGTTGGTTAA CGCTTCGCCA AACGCAATGA
781 TCGCAGCGCG TGACGCTTCG CCACCTGTTC GGCACCGGTG TAAGCACTGT AAGCGCCATT
841 TTGCGTTTGG TTTTTCGTTT GGCGTTTGGG CGGAGGTTTG GCGCGAGTGT GTATTGTAGA
901 TAGTGCCGAA CGGCAGCTGT GGGAGCACTC GTAGATACAG AACCGTTTAT CATCAAGTCG
961 TGGAGGGCAG CGGCGCCCTC TTTTGTTGCT GCTCTTCGTC

DDB2

1 GTGTTTGTCA AGCGCTGTCG GAATGTTCTG AACGAACAAG ATACGATTCG GGACTGCGGG
61 AGCAGCGATG GGGTGTGGTA CGATGGCAGC CACGATGGGA GGTGCATTCG GCATAGCAGT
121 CGACGACGGG ACAGCAGTGG AAGCAAGAGC ATGTTGCTGC TGTTGCTGCT CCGCCGCGGC
181 GATGTGGTCG CGCTTGACAG CGAGGGCATC GGCGCGACGT TTGGCAGCCC GTTTGTTTCT
241 AGAAAGCGTT GAATCGCCGC CCAGTTTTTC GCTGGCGGCC TTGTCGGAAA GAGCTCTGGC
301 GTAACTGACG GAGATGTGGT TGCCCATGAA CTCGGAGGCG TGGAGCTTTC TGAGCGCGGC
361 TGTGGCGGAC GCCTGGGTGT GAAAGGTGAT GAAAGCTTGG CCGCGAAGCG GAATGGACTT
421 CTCGGCCACA ATGTGCACGA CGCGTCCGTA CGGACTGCAC GCGGCGTGCA GCAGGCGACG
481 AAGGCGGTGC TTCTTGAGCT TGTCCGGCAG GTTACGCACG TACAGCGTGC GGCAGGGGGC
541 CGGTGCGTCC ATGGCGACGA CTCGGCGACG ACTCGGCGAC GACTCGGCGA CGACTCGGCA
601 ACGACTCGGC AACGACTCGG CAACGACTCG GCAACGACTA AGCAACGACT CGGCAAGGCG
661 GGCGCGCGCG TTTCGCTTCG CGCCGCGGCG ACGCCACTTG CGTTTGGTAG CCGACCGCCG
721 GAGCGGCGTT TGGTAGCCGA CCGCCGGAGC GGCGAAGCGA ATTTTTGGCG TTTCGCTTCG
781 CGCGCGGCGA CGCCACTTGC GTTCGGTAGC GGGGGGGGGG TCAGCNTTTA TTGTGGTCAC
841 TGTCCCGGTA GCCGATCGCC GCAGCGGCGA AGCGAAACGC GTCACGACGG CTGTCACGCG
901 CACACGTGTG GCGTTTTGTG TTCGCGAGAA GCCAAACGCA ACGCGCGCGT CGCCGCCAAC
961 GTCGCGCACA GCGCCAGCAC CGCCGCCCCA TCCCGCCGCC

PIAS1

1 GCGACGAATG TGACTGAGGC CTTTTGTAGA CAGCATACGG TCGAAACCTG TACGCCTTCT
61 TCACGAATCC TCAACCCACT AAAGTCAAAT TCATTGATGC CCAGGCTTTT CTTCCATCGA
121 CGCCACTGCG GTGCACAGAA GGAGCCCGCA CCCACTTCCT CGTCCCAGAT AAAGGAAAAG

181 AGTACGTTAC ACTCGGCCTG CCGCTGCCAT TCAGGAAAAC AATCTCGCCC ACTTCGCGTC

241 TTCTGCATTC CTTGCGTCTT CGTCAGTATC AAGGATGTGC ACGCAGCTCA CCCTTCTCCC

301 AACTGTGACT GCCAGTGTAA GTCTGCATTC AAAAGTACAA CTCCGGAAGT TTCGAATACG

361 TCCCCGGTAC AGATGTCTGC CACCACGGAC ATGACTCCAG CCAATGTTCC GACTCCTCAT

421 TTGCCTGGTC GAATTATTCC GGTTGCGCAG CATATTCAGG AAAGCCAGAA CCAAGGAACC

481 GATCAAACAG CTTCCAATCT TAGACGAGTT GCACCACATG TCAGTGGTAA CACGGATGCA

541 AATCGTCGTT CAGCCACTAT ACCATCGTCC ATGCTACATG ATGTCAACGC GAATATGACT

601 GTTGTATTGA ATTTGAGTGA AGCCGGGCAA ACTATATATC CGCCATATCT TCCAGACGGT

661 AGAGTTGAAC ATGCCCTCAC GCCGAGAGAA GTGGATTCTC CCAATATGGC TAGCGATAAC

721 CGCAGTGACC AGCATCCTTC GTCTCGTTCG TATTCTACTT CTGAGTCTGG AGATGTATTC

781 GAGGAGGACA GTGATGTTAC GGAAGAGACT TCCTACGACA CGCTCGTCAA ACAATTACAA

841 CAAAGTAAGT TCTTACCATT CTTTGCGGTG TCTTCCATAT TCATGCCTCC AGGATTGCAA

901 TTCAAGTTGC TAACTCTCTC TAGCTTCACC GGAAGCGATT TCACGCACCA TACAGGCTGT

961 AAAGGCGAAA GACCTTTCTA ATGATGCACG TTCTAAACTA

FZR1

1 TTATCAATGT ACTTGAAGAC GTCTGCGAAT ATGTCGGGGG TAGATTTCTT GTAGTGCGGA

61 GTTGCAGATG GATAGCGCTT TGAGTCGATG CCGCCGTTGC CGGGAGATGC ATGGACGCAG

121 TTGTGGATGA TGCCGTTCAT GTCGAGATAC AAGTTGTCGA TTTTGGGGGC GGAGTTATGA

181 CGTAACGGGG CGTTGATGAG GGGGTAGCGC TCGCTGATGT AGCGGTAGAA CTTTGGAACT

241 CCTGCGGGGT GTGTACGAAA GCGCAATTGG ATTGCGGAGA TGGGGGGAGG GTCAGAGAGA

301 TGCTGGAGAC GGAAGGGCAA ACGCGGACGC AAGCACGGGA AGAAAGCACA GACGTACCCA

361 TGGTGGGGGG ATGCGGAACT GGAAGGGGGG AGAGAGGGGG GGAAAAGGGC GGAAGGGGTT

421 CACGGGGCTG TCAACGTCGG GAACGCAGCA GCGTACTCTG TAATAATGCA GCAGGTGGGA

481 GAGGAGCTGA GGTAAGCCAA CAGCGTACAC TATGTAGGGT AGAGAAGTAC ACGCAAGGCG

541 CGACACAGAA AGGACGCTGA AGCGAAAGCC GTGACAGCAG TTTGCATTGG CGAAGCGGCG

601 CAGCACAGCA CAGCACAGCA CAGCACAGCG ACAGCGCGCC GCCTGGCTTA TCCTCGCGCC

661 GCTGCTTTCG TTTCCACTTC TCAGCGCAGA CCCAGAAACG GAATGCCACG TGTACCACGC

721 ACACACGTGT GTTGGTGGAG GTGTGCGCAT CGGGTGGCAC CGCGCCCCCG ATGGCGCCAA

781 ATCATGCCAA GTCACGCGCG CGCACGCTCG CCGGCACGAT GCGTCGCCCT CGTCGCGCGC

841 CGCTTCCGCT TGCGGAACTC CCGCGCGCGT CGTCTTCCCT CCGCCCCCGC GTCCCGCTCC

901 CTACTTGAGA CCGCTCGCCA TCTCCGCCCC GACAGCGCCA CCCTCTCTCC AGCCCCCCAA

961 ATCCTCATCC CTCTTCCCCG ACGCCCTTCT CCCACAAGCC

APC8

1 ACTACTGATA TGCTCACGGG TATGTGTGTA GGTATGTTTC TGGGCGTGGC TGCTTTTTCC

61 GAACACAAGG TAGAGCTTCT TACGAAAAGA AGCTCCCTCT GTCAAGGCAG GACAGCACTC

121 GCTCCACGTC CAAAACCGCT AACCAATATG CCCTACTGAG GCCGTAGTAG TTCAAACCTT

181 CTCAATAGCG CCAGCAAGCG GCACCGAAGC AGACAGTGAC TTGCGCTATT CGTAGCGTTC

241 GGCATGCATG TTGATGGGCG CACTGCCTTT TCGTTGTTAA GATAGGTTGC AACCGGATAC

301 AACATAAGTG ATTGTTTAAC TCCCAGCTGA AATGAGGCTA CAATGAAACC AGGGACGTTT

361 GCGCCTGACG CAACATAGTT TTCCTTCAAG TAAGTTTGGT GGACTTGCTA TTTCTGCCCT

421 TCTTCACGTG CTGTTCCTTA TCCTCCACGG GCATGAGTGC TGCTGTAGCG GCTGCGGGCG
481 GTGTCGTCGA AGTAGAATGG GGGACTATTC GACAACATGC AAAGTTCGCG TCTATAACAT
541 TGGGGGCGTT GGAGGATACG GATGATAAGA ACCCTCACGG ATGTAGATTT GTGTGACGAC
601 GATCATCGTT CATGATGAAG TTGGCGGTCT TGCAATCTAG AAGCTTGACA GACAACTCCC
661 ACCAGCGGAG AAACTATGAC AGGAATTTGC AGGATAACTT TTAAATACAG AAAACATGCT
721 TCGTGGGACA GTCCTTTTTG CGTACAAGGG ATCAGCACAA ATTCGATTTG CGCTTAAGCT
781 TCGGGAAAGG GAGCACTAAA GCACCTGCCG TTAATTGTAA GACACCGTTA TGCTCTGTTA
841 CGAACCGTCC GTCTGCGGGA GATACGCCTT TCGCTGCGTC TGAACACCTT CATCGATCGT
901 GTTTCCGACG AAAGAATCGT GTCAGAAGAG TGCTATGCAT CACAGACCTA TTCAGTGACG
961 CCGTATACAC AAGTTGTTCC CCTCAGTTTG CGCCGCAGAG

COP1

1 GAGGTGAGCG GAGAAGAGTG AGCGCAAAGA GTTCGCAAAC ATCAGATCCG CGCAATTTAG
61 GTAGTGTGCG GGAAGCGAGG AATCACGCCA CGAGCCAAGG AGAACCCGCC AACGTGTTAC
121 TACGAACAGG GACAGGAATT CTTGGAAAGG TCATTTCCAT CCTGAACGAA GTGCGCACTA
181 CAGATCAGAA TGACACTCTG AAGTCCAGGA TCGACGAGTC AACCAGTAGC TTGGAGTCCG
241 CGATAGGACA GCAGAGAGAA GACCAAGAAT CCGGTAGGGG CGGGGGCGGC GGGGTGCCAG
301 TATAGTGCGT CGGATGCCTA TGGTTGGACC TAGAGGACGA AGACGGGGGC GGGGGCGGCG
361 GGGTGCCAGT ATAGTGCGTC GGATGCCTAT AGTGGACGCT AATGAGGACT AATACGTTTC
421 GGCGGCGGCG GGTGCTCAGT ATAGGTGCGT CGGATGCCTA TAATGTTGGA CGGCTAGAGG
481 CGAAGACGGG GGCGGGCGGC GGCCGGGGTG CCAGTATAGT GCGTCGTGAT GGCCCTATTA
541 GTTGGAACCC TAGAGGACGA AGACGAGGGC GGCGGCGGCG GGTGCCAGTA TAGTGTCGTC
601 GGATGCCTAT AGTGTGGAAA GCTAGAGACG AAGACGAGGG CGGCGGGCGG CGGGGCTGGC
661 CAGTATGTGG CGTCGGATGC CGTATAGTTG GACGCTAGAG GACGAAAGAC AGGGCCGGCT
721 GCCAGTATAG TGCGTCGGAT GCCTATAGTT GGGCGGCGGC GGCGGCGGGG TGCCAGTATA
781 GTGCGTTGGA TGCCTATAGT TCGGGGGCGG GGGCGGCGGG GTGCCAGTAT AGTGCGTCGG
841 ATGCGTATAG TTGGACCTGA TCACGCTTGG GCTCAAGGCG GCGCGCGCGG GTGCCCAGTA
901 CCCATTCTCC AGTGCGTCCC GAGCGTGCCA TGGGTTCTCG GCACCAACTA CGGTGTGACC
961 TCCGAGCTCG TCGAAGCACA GCTCCCGGGC GGCGCGGGCC
